# Sexual Dysfunction in Patients with Overactive Bladder Syndrome Treated with Botulinum Toxin

**DOI:** 10.3390/jcm13195869

**Published:** 2024-10-01

**Authors:** Joanna Sondka-Migdalska, Pawel Blaszczynski, Zbigniew Jablonowski

**Affiliations:** 1 1st Department of Urology, Medical University of Lodz, 90-549 Lodz, Poland; 2 Psychology Department, Pirogow Regional Hospital in Lodz, 90-001 Lodz, Poland

**Keywords:** overactive bladder (OAB), quality of life, sexual function, botox, onabotulinumtoxinA

## Abstract

**Introduction:** Overactive bladder (OAB) is a syndrome of the lower urinary tract characterized by urinary urgency, frequency, and nocturia, with or without urgency urinary incontinence. OAB significantly impacts all aspects of life—social, psychological, physical, professional, domestic, and sexual—for both women and men. The aim of this study was to investigate sexual dysfunction in both women and men with OAB treated with intravesical onabotulinumtoxinA (Botox) injections using the Sexual Quality of Life questionnaire in two versions: female (SQoL-F) and male (SQoL-M). **Methods:** Forty sexually active patients (thirty women and ten men) with idiopathic OAB were recruited. Patients completed the SQoL-F or SQoL-M questionnaire before treatment, and again at 3 and 6 months after treatment with intravesical onabotulinumtoxinA injections. **Results:** All 40 patients completed the study (30 women and 10 men). There were no statistically significant differences in SQoL results before the procedure or at 3- and 6-months post-treatment. **Conclusions:** OAB treatment with onabotulinumtoxinA did not significantly affect the quality of sexual life in either women or men. Further research is needed using questionnaires specifically designed to assess the sexual life of patients with OAB, especially in men.

## 1. Introduction

Overactive bladder (OAB) is a syndrome of the lower urinary tract characterized by urinary urgency, frequency, and nocturia, with or without urgency urinary incontinence, in the absence of urinary tract infection or other pathology [1,2,3]. One of the largest epidemiological studies, EPIC, showed that OAB affects as many as 11.8% of the population (10.8% in men and 12.8% in women) [4]. The study conducted by Milsom in Europe indicated that overactive bladder syndrome occurred in an average of 16.6% of the population (17.4% women, 15.6% men) [5].

In the course of OAB, the gradual intensification of symptoms usually leads to lifestyle changes and may significantly affect the quality of life [6,7]. Increased interest in this topic has revealed the enormous impact that OAB can have on social interactions, sleep, depression, sexual health, and overall health-related quality of life (HRQoL) [8]. OAB strongly affects overall HRQoL, including the physical, mental, and sexual health of patients.

The main cause of OAB is not yet known. Neurological disorders and changes in the lower urinary tract are considered, but the most common cause is idiopathic.

Diagnosis of OAB is based on patient history and the exclusion of local factors that may cause symptoms of overactivity. Many organic, functional, or even pharmacological factors can cause symptoms of overactive bladder. Thus, to diagnose overactive bladder syndrome, we must exclude, among others, urinary tract infection, bladder cancer, bladder lithiasis, neurological disorders, foreign bodies, and pelvic irradiation [2,9].

Three groups of treatment methods are used in OAB therapy: conservative methods, pharmacotherapy (anticholinergic and beta-adrenomimetic drugs), and surgical methods—intravesical injection of botulinum neurotoxin type A into detrusor wall, sacral neuromodulation [10]. The treatment of OAB aims to improve the quality of life for patients experiencing symptoms of varying severity. Therefore, it is essential to determine the cause to select the most appropriate treatment and establish a therapeutic goal. Treatment is always tailored individually, starting with the least invasive and progressing to more invasive therapeutic methods [9,10].

On 18 January 2013, the U.S. Food and Drug Administration (FDA) approved an expanded indication for Botox (onabotulinumtoxinA). This approval was based on two clinical trials involving 1105 patients exhibiting symptoms of overactive bladder (OAB) [11]. Botulinum toxin is a neurotoxin that inhibits neuromuscular transmission. Intravesical (submucosal) administration of botulinum toxin reduces intravesical pressure, thereby increasing bladder capacity and significantly decreasing urinary frequency, urgency episodes, and incontinence [12,13].

According to the World Health Organization (WHO), sexual health is defined as a state of physical, emotional, mental, and social well-being related to sexuality. Sexual health involves experiencing pleasurable and safe sexual experiences, free from coercion, discrimination, and violence [14]. Assessing sexual quality of life is essential for evaluating the quality and prevalence of sexual disorders [15].

Sexual dysfunction is defined as a frequent and repeated inability to participate in sexual relations in accordance with a person’s desires, lasting for many months. Sexual dysfunctions can be divided according to the phase of the sexual reaction cycle they affect and those related to the feeling of pain.

1. Desire phase: lack or loss of sexual needs (decreased sexual drive), excessive sexual drive.
2. Arousal phase: erectile dysfunction (men) and lubrication disorders (women).
3. Orgasmic phase: delayed or absent orgasm in women, premature ejaculation or delayed ejaculation in men.
4. Pain disorders: vaginismus (women), dyspareunia (both sexes) [16,17].

## 2. Materials and Methods

The aim of this study was to investigate sexual dysfunction in both women and men- with OAB treated with intravesical onabotulinumtoxinA (Botox^®^, Allergan, Inc., Irvine, CA, USA) injection using the Sexual Quality of Life questionnaire in two versions: female and male (SQoL-F, SQoL-M).

In this study, we use the ISC definition of overactive bladder syndrome (OAB), which is “defined as urinary urgency, usually with urinary frequency and nocturia, with or without urgency urinary incontinence” [18].

Inclusion criteria:

– OAB resistant to pharmacological treatment (3–6 months of pharmacotherapy without effect).
– Correct general urine examination.
– Sterile urine culture.
– Bladder diary.
– Idiopathic overactive bladder confirmed by urodynamic study.
– Ultrasound of the urinary system with assessment of post-micturition retention.

Urodynamic testing is mandatory before any invasive treatment. All patients included in the study underwent urodynamic testing, and none of the patients showed.

Each patient underwent urodynamic tests prior to invasive treatment. These tests included a medical history, physical examination, bladder diary, uroflowmetry with post-void residual urine assessment, and cystometry. None of the patients qualified for the procedure showed neurogenic detrusor overactivity or bladder outlet obstruction in the conducted urodynamic tests.

Exclusion criteria:

– Urinary tract infection (UTI).
– Lower urinary tract urolithiasis.
– Bladder cancer.
– Post-void residual urine.
– Pregnant women.
– Patients after pelvic radiotherapy (RTX).
– Bladder outlet obstruction Benign prostatic obstruction (BPO).
– Previously diagnosed interstitial cystitis/bladder pain syndrome (IC/BPS).

The study was completed by 40 patients (30 women and 10 men) aged 35 to 78 years, with a mean age of 58 years. Patients with idiopathic OAB underwent a procedure involving the administration of botulinum toxin into the bladder, all performed by a single operator. A single dose of antibiotic (cefazolin iv) was administered 30 min before the procedure. During cystoscopy under short-term intravenous anesthesia, a flexible 6F needle was used to inject the prepared solution of “Botox” (Botox^®^, Allergan, Inc., Irvine, CA, USA)—100 units of botulinum toxin dissolved in 10 mL of 0.9% NaCl. The solution was administered in 20 injections of 0.5 mL each into the detrusor muscle, targeting the posterior and lateral walls of the bladder while avoiding the trigone area.

Patients completed the SQoL-F or SQoL-M questionnaire before as well as 3 and 6 months after treatment with intravesical onabotulinumtoxinA injections.

The Sexual Quality of Life-Female (SQOL-F) questionnaire is a concise instrument designed to assess sexual self-esteem, emotional issues, and relational aspects. It comprises 18 items, each rated on a six-point Likert scale ranging from “completely agree” to “completely disagree”. The response categories can be scored either from 1 to 6 or from 0 to 5, resulting in a total score range of 18–108 or 0–90, respectively. Higher scores indicate a better sexual quality of life for females [19].

The SQOL-M is a modified version of the SQOL-F, specifically designed for men. This questionnaire comprises 11 items, with response options identical to those in the SQOL-F version. The total score ranges from 11 to 66 or 0 to 55. Higher scores indicate a better quality of sexual life for men.

To transform the raw scores onto a standardized scale of 0 to 100 for easier comparisons with other measures, you can use the following formula:

$$\mathrm{Scale}\;\mathrm{Score}=\frac{\mathrm{sum}\;\mathrm{of}\;\mathrm{the}\;\mathrm{component}\;\mathrm{items}\;\mathrm{minus}\;\mathrm{the}\;\mathrm{lowest}\;\mathrm{possible}\;\mathrm{score}}{\mathrm{possible}\;\mathrm{raw}\;\mathrm{score}\;\mathrm{range}}\times 100$$

Higher scores indicate an improved quality of life. For questionnaires with missing responses, a total score can be calculated for an individual if at least 50% of the items (minimum of 6 items) have been completed. Questionnaires with more than 50% missing items should be excluded from any analyses [19].

The main reason for using this questionnaire is that it is widely available, easy to use, and understandable for patients, and it does not suggest sexual dysfunction. Additionally, an important factor is that the questionnaire has a similar format for both genders. The SQOL questionnaire has been demonstrated to be the most accessible and appropriate for patients with OAB [20].

A total, all 40 patients were approached and agreed to complete the questionnaire and finish the research. The baseline characteristics of the study participants are shown in Table 1.

All subjects gave their informed consent for inclusion before they participated in the study. The study was conducted in accordance with the Declaration of Helsinki, and the protocol was approved by the Ethics Committee of District Medical Chamber of LODZ (KB -10/16 consent obtained on 11 May 2016). 

The obtained research results were subjected to statistical analysis. The values of the analyzed quantitative parameters were presented using descriptive statistics: standard deviation, mean value, median, minimum and maximum values. The level *p* < 0.05 was considered statistically significant. All calculations and estimate statistics were performed using the Statistica™ program, version 13.3 (TIBCO Software Inc., Palo Alto, CA, USA).

## 3. Results

In total, 40 patients completed the study. There were no statistically significant differences in SQoL results before the procedure, after 3 months, and after 6 months (Table 2).

The treatment of overactive bladder (OAB) with botulinum toxin did not significantly improve sexual function in the studied women and men, but it is important to emphasize that it absolutely does not worsen it. The results of this study suggest that if the treatment dedicated to the underlying disease does not significantly affect the sexual function of the patients, it may indicate that OAB may not disrupt this aspect of life.

In terms of adverse effects associated with intravesical botulinum toxin A therapy, our patient cohort did not experience any instances of urinary tract infections. Additionally, none of the patients required clean intermittent self-catheterization due to urinary retention or increased post-void residual urine volume.

## 4. Discussion

Sexuality is a challenging aspect of life to examine because patients often do not seek help when they have a sexual disorder. Patients are reluctant to admit to their disorders due to feelings of shame, fear, and embarrassment. There are few studies in the literature regarding communication about sexuality. Research has shown that as many as 69% of psychiatry residents feel discomfort or avoid questions about sex [21,22].

It is also important to emphasize the significance of overactive bladder (OAB) and the accompanying psychological aspects of this condition. Brodsky highlights in his research that OAB in patients causes significant stress that extends beyond physical symptoms; it leads to feelings of isolation from friends and family and makes them feel embarrassed when discussing their condition with relatives and physicians [23].

Hawken et al. confirm in their study that up to 80% of surveyed patients report embarrassment and shame associated with OAB symptoms—incontinence, frequency, or nocturia. They also reported fear, fatigue, stress, anxiety, and anger caused by OAB. There were patients who waited 17 years to talk about incontinence with their general practitioner due to embarrassment, shame, and worry [24].

In this conducted research, two sensitive areas of life were addressed and combined. To date, no research has been conducted on sexual dysfunction following the administration of botulinum toxin in patients with overactive bladder syndrome (OAB) using the validated Sexual Quality of Life (SQoL) questionnaire for both women and men [4]. In Poland, SQoL for both women and men has been used in several studies so far [25,26,27].

Most studies report that OAB negatively impacts the sexual quality of life for women, and various treatment methods can improve it. In a meta-analysis published by Evans in May 2024, it was demonstrated that pharmacological treatment of OAB with anticholinergics and beta-3 agonists can significantly improve sexual function in women [28]. However, it also highlights that the topic of sexuality in patients with OAB is still overlooked in everyday practice.

There are, however, reports that do not show a connection between improved sexual function and OAB treatment. Jha, in his study, demonstrated a positive impact of pharmacological treatment of OAB on sexual function in only 8% of those recruited, compared to 66% of patients who experienced relief from OAB symptoms [29].

Miotła et al. demonstrated a significant impact on the improvement in sexual quality of life in female patients with OAB treated with botulinum toxin, using the FSFI questionnaires. However, it is emphasized that their studies are still insufficient due to the small number of subjects. Their study was the first published to demonstrate a connection between sexual life and OAB treated with botulinum toxin. They highlight the small number of subjects and suggest further research on this issue [30].

Further studies were conducted by Balzarro et al., who examined 32 female patients with OAB who underwent intravesical botulinum toxin treatment and observed an improvement in sexual function and performance. They also emphasized the necessity of conducting broader studies on a larger group [31].

The study conducted by Giannantoni et al. evaluated the effects of intravesical onabotulinumtoxinA injection on sexual function in patients diagnosed with overactive bladder (OAB) syndrome and multiple sclerosis (MS). Despite involving a distinct patient cohort, the decision was made to include the study due to its comprehensive analysis of sexual function scores before and after Botox treatment, reflecting the treatment’s impact on sexual function. Urinary disturbances and sexual dysfunction significantly reduce the quality of life and impact the psychological state of patients with multiple sclerosis (MS) who experience low disability levels. Intradetrusorial injections of onabotulinumtoxinA not only manage urinary symptoms in MS patients but also lead to a marked improvement in their sexual functioning [32].

In 2008, Irwin et al. conducted a secondary analysis of the EPIC study to assess the prevalence of self-reported erectile dysfunction (ED) in men with overactive bladder (OAB) compared to a control group. This analysis included 502 men with OAB and 502 controls. ED was evaluated using a single-question format. The findings revealed that men with OAB were significantly more likely to report ED compared to controls [33].

On the other hand, Przydacz noted that OAB did not significantly affect men’s sexual activity in terms of the frequency of sexual intercourse and the number of sexual partners. They were the first to conduct such a study on such a large group assessing the impact of OAB on the sexual life of men [34].

Truzzi demonstrated in his analysis that there is still insufficient data to definitively assess the impact of OAB treatment on sexual life. On the other hand, Levy et al. showed that the treatment of OAB in women does not have a negative impact on sexual function, which also supports our findings—this applies to both women and men [35,36].

The small number of patients participating in the study suggests a difficulty in encouraging participation in the survey. In the presented study, 40 patients were included. The limited number of patients is due to the numerous inclusion and exclusion criteria for the study.

No impact of OAB treatment on the quality of sexual life has been demonstrated in women or men. Further studies using questionnaires specific to the assessment of sexual life in OAB patients, especially in men, are needed. The current state of the literature does not provide a basis for drawing definitive conclusions. In surveys, it is worthwhile to consider whether issues occurring in OAB, including urinary incontinence, affect the sexual sphere of patients—using questionnaires for women and men that can be compared.

## Figures and Tables

**Table 1:** Baseline characteristics of the study participants (*n* = 40).

Analyzed Trait	Statistical Parameter *
	*n* (%)/*M* (*SD*)
**Sex:**	
- **Female**	30 (75.00)
- **Male**	10 (25.00)
**Age (year)**	58.26 (13.71)
**Body weight (kg)**	79.77 (16.55)
**Height (m)**	167.55 (9.04)
**BMI (kg·m^–2^)**	28.40 (5.24)

* For discrete variables: *n*—number, %—percentage. For numerical variables: *M*—mean, *SD*—standard deviation. BMI—body mass index.

**Table 2:** Descriptive statistics for the SQoL scores in the study participants by gender.

Gender	Time Point	Statistical Parameter *				
		*M*	*SD*	*Me*	*Q*_1_–*Q*_3_	
**Female**	**Before treatment**	40.69	27.79	33.33	25.56–51.11	= 0.3817
	**After 3 months**	47.41	29.88	50.00	25.56–66.67	
	**After 6 months**	46.04	28.77	44.44	25.00–62.78	
**Male**	**Before treatment**	48.28	35.58	63.64	21.82–69.09	= 0.9370
	**After 3 months**	49.09	28.30	44.54	21.82–74.54	
	**After 6 months**	52.27	28.96	46.36	24.54–82.73	

* *M*—mean; *SD*—standard deviation; *Me*—median; *Q*—quartiles.

## Data Availability

The original contributions presented in the study are included in the article, further inquiries can be directed to the corresponding author.
